# Recent progress in the development of solid catalysts for biomass conversion into high value-added chemicals

**DOI:** 10.1088/1468-6996/16/3/034903

**Published:** 2015-05-20

**Authors:** Michikazu Hara, Kiyotaka Nakajima, Keigo Kamata

**Affiliations:** 1 Materials and Structures Laboratory, Tokyo Institute of Technology, Nagatsuta-cho 4259, Midori-ku, Yokohama 226-8503, Japan; 2 Frontier Research Center, Tokyo Institute of Technology, Nagatsuta-cho 4259, Midori-ku, Yokohama 226-8503, Japan; 3 Japan Science and Technology Agency (JST), Advanced Low Carbon Technology Research and Development Program (ALCA), 4-1-8 Honcho, Kawaguchi 332-0012, Japan; 4 JST, Precursory Research for Embryonic Science and Technology (PRESTO), 4-1-8 Honcho, Kawaguchi 332-0012, Japan

**Keywords:** biomass, heterogeneous catalyst, solid catalyst, zeolite, metal oxide, metal hydroxide, supported nanoparticle

## Abstract

In recent decades, the substitution of non-renewable fossil resources by renewable biomass as a sustainable feedstock has been extensively investigated for the manufacture of high value-added products such as biofuels, commodity chemicals, and new bio-based materials such as bioplastics. Numerous solid catalyst systems for the effective conversion of biomass feedstocks into value-added chemicals and fuels have been developed. Solid catalysts are classified into four main groups with respect to their structures and substrate activation properties: (a) micro- and mesoporous materials, (b) metal oxides, (c) supported metal catalysts, and (d) sulfonated polymers. This review article focuses on the activation of substrates and/or reagents on the basis of groups (a)–(d), and the corresponding reaction mechanisms. In addition, recent progress in chemocatalytic processes for the production of five industrially important products (5-hydroxymethylfurfural, lactic acid, glyceraldehyde, 1,3-dihydroxyacetone, and furan-2,5-dicarboxylic acid) as bio-based plastic monomers and their intermediates is comprehensively summarized.

## 1. Introduction

### 1.1. Role of solid catalysts in green and sustainable chemistry

Although the petrochemical industry has contributed to worldwide economic development over the past century, many serious environmental problems still sometimes arise. Therefore, to establish environment-friendly chemical processes requires the development of novel and cost-effective approaches to pollution prevention. Green chemistry is one of the most attractive concepts for pollution prevention, as developed by Anastas and Warner in their 12 principles [1]. A brief definition is that green chemistry reduces and/or eliminates the use or generation of hazardous substances in the design, manufacture, and application of chemical products. Advances in green chemistry will contribute to the abatement of obvious hazards associated with global issues such as climate change, energy production, availability of safe and adequate water supplies, food production, and the presence of toxic substances in the environment. The challenges faced to achieve sustainability will lead to new technologies that provide society with products that we can depend on in an environmentally responsible manner [1–4].

For the manufacture of chemicals (especially high value-added chemicals), antiquated stoichiometric technologies are still widely used, such as acid/base-catalyzed reactions with mineral acids (H_2_SO_4_, H_3_PO_4_, etc), Lewis acids (AlCl_3_, ZnCl_2_, etc), and inorganic bases (NaOH, KOH, etc), reduction with metals (Na, Mg, Fe, Zn) and metal hydrides (LiAlH_4_, NaBH_4_), and oxidation with permanganate or chromium(VI) reagents. However, these stoichiometric reagents cannot be recovered and recycled. Therefore, the use and generation of toxic and hazardous substances can be reduced by replacement of these stoichiometric methodologies with cleaner catalytic alternatives [1–7]. Many soluble homogeneous catalysts have been developed to date for the green synthesis of various chemicals. These catalysts are usually dissolved in reaction media, which makes all catalytic sites accessible to substrates, and exhibit high catalytic activity and selectivity. Despite these advantages, homogeneous catalysts have a share of only ca. 20% in industrial processes because catalyst/product(s) separation (i.e., product contamination) and reuse of expensive catalysts are very difficult to achieve efficiently and cost-effectively [8]. In this context, the development of easily recoverable and recyclable solid catalysts has received particular interest for environment-friendly syntheses of high value-added chemicals.

**Figure 1.**
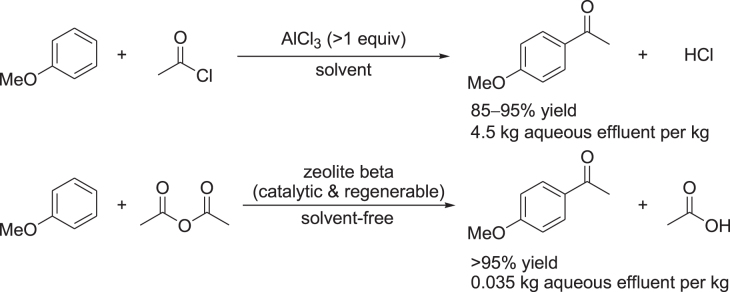
Comparison of the AlCl_3_- and zeolite-catalyzed Friedel–Crafts acylation processes [7].

As an example, the AlCl_3_- and zeolite-catalyzed Friedel–Crafts acylation of anisole are compared (figure 1) [7]. Zeolite-catalyzed acylation uses acetic anhydride as the acylating agent and requires no solvent. In contrast, the AlCl_3_-catalyzed process uses acetyl chloride and requires more than one equivalent of AlCl_3_ (the use of anhydride would require >2 equivalents of AlCl_3_) and a chlorinated hydrocarbon solvent. Thus, the zeolite-catalyzed process avoids the generation of HCl and the use of acetyl chloride for synthesis. In addition, the zeolite-catalyzed process has the following advantages: (i) the amount of aqueous effluent is >100 times less than that for the classical AlCl_3_-catalyzed process, (ii) a higher chemical yield of *p*-methoxy acetophenone is achieved, (iii) the catalyst is recyclable, and (iv) the number of unit operations is reduced from 12 to 2. In this context, heterogeneous catalysis can be expected to remain a cornerstone in building a sustainable chemical community through green chemistry [8–13].

### 1.2. Biomass conversion into value-added chemicals and fuels

**Figure 2.**
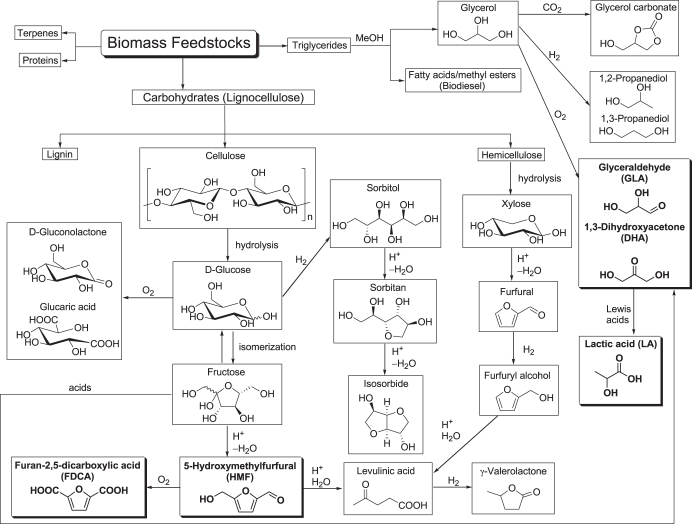
Representative processes for biomass conversion into chemicals and fuels.

There has been continued focus on waste minimization and the avoidance of toxic and/or hazardous reagents and solvents, particularly in the fine chemicals and related industries, as discussed in section 1.1. In recent decades, the substitution of non-renewable fossil resources such as crude oil, coal, and natural gas by renewable biomass including lignocellulose and triglycerides as a sustainable feedstock has been extensively investigated for the manufacture of high value-added products such as biofuels, commodity chemicals, and new bio-based materials such as bioplastics [14–53]. Representative chemocatalytic processes for the manufacture of such high value-added products are summarized in figure 2.

**Figure 3.**
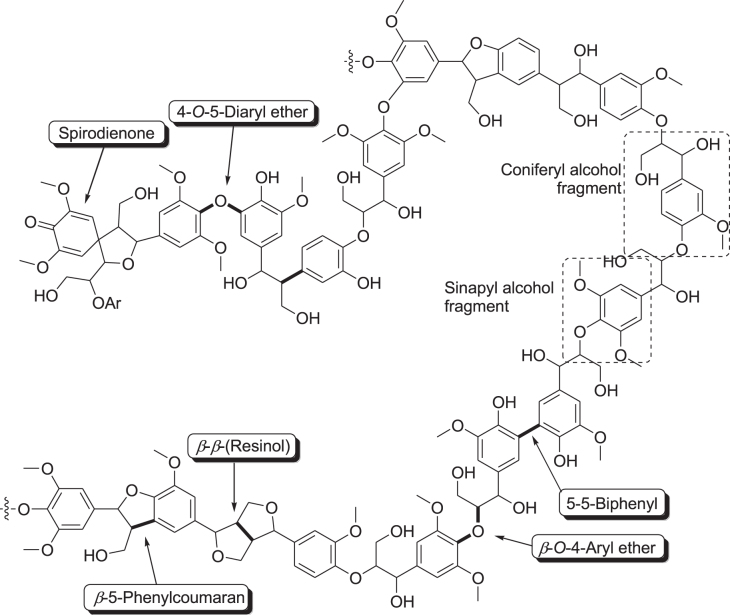
Schematic representation of a hardwood lignin structure and common linkages found in the lignin polymer [40, 41].

In contrast with edible biomass such as starches, sugars, and vegetable oils, the most abundant lignocellulose, the fibrous material that constitutes the cell walls of plants, is an inexpensive nonedible biomass that could be an excellent source of fuels and chemicals without affecting food supplies. Lignocellulose is composed of ca. 20% lignin, ca. 25% hemicellulose, and ca. 40% cellulose [14–32]. Lignin is a three-dimensional polyphenolic biopolymer with a non-uniform structure that imparts rigidity and recalcitrance to plant cell walls (figure 3), but its selective conversion into chemically useful aromatic components remains a challenge [40–42]. The hemicellulose polymer is formed by pentose and hexoses (e.g., D-xylose, D-galactose, D-arabinose, D-glucose, and D-mannose), of which xylose is the most abundant [38, 39]. Cellulose is also a polymer of glucose units linked by *β*-glycosidic bonds [37]. While lignocellulose is abundant as a resource from plant material, its exploitation has been limited due to its composite nature and rigid structure. Therefore, pretreatment to liberate cellulose and hemicellulose from the lignin composite is required.

The three main catalytic routes used to transform biomass into fuels and chemicals are gasification, pyrolysis and hydrolysis [33–36]. In this review article, we mainly focus on hydrolysis processes used to break the lignocellulose into its constituent parts, and hydrolysis-related processes. The cellulose and hemicellulose can be hydrolytically converted into their constituent building blocks, such as C5 and C6 monosaccharides, and these pentoses and hexoses can be subsequently used as raw materials for chemocatalytic conversion processes to biofuels or platform commodity chemicals such as polyols, furans, and acids (figure 2) [37]. Cellulose is hydrolyzed into glucose monomers under severe reaction conditions (i.e., at high reaction temperature with sulfuric acid as a catalyst). Furthermore, glucose can be converted into various useful chemicals by oxidation, hydrogenation, and dehydration. The oxidation of glucose affords gluconic acid, which is successfully oxidized to glucaric acid as a monomer for the preparation of biodegradable polyamides with unique properties. Glucose is hydrogenated with H_2_ to form sorbitol, followed by acid-catalyzed dehydration to sorbitan and subsequently isosorbide as an industrial monomer. Acid-catalyzed dehydration of fructose, which is formed by the isomerization of glucose, gives 5-hydroxymethylfurfural (HMF) as a raw material for the production of chemicals, polymers and biofuels [52, 53]. However, to achieve commercially viable selectivity toward HMF is a challenge because of the propensity of HMF towards further reaction under the acidic reaction conditions. HMF is oxidized to furan-2,5-dicarboxylic acid (FDCA), which is a structural analogue of the terephthalic acid monomer. Polyethylene furandicarboxylate can be an alternative bio-based plastic to replace polyethylene terephthalate. Further reaction of HMF with water under acidic conditions yields levulinic acid with the concomitant formation of formic acid. Levulinic acid is an attractive platform chemical itself and subsequent hydrogenation affords *γ*-valerolactone, which is a potential building block for polyesters. A polymeric xylan, which is obtained from the hemicellulose fraction, can be depolymerized to form a xylose monomer through diluted acid hydrolysis (e.g., with sulfuric acid) [38, 39]. Pentoses including xylose can also be converted to levulinic acid. This process involves the dehydration of xylose to furfural and subsequent hydrogenation to furfuryl alcohol, which is finally hydrolyzed to levulinic acid.

In addition to lignocellulose, conversion of triglyceride feedstocks (e.g., inedible plant oils, algal oil, or waste cooking oils and fats) into biodiesel and platform chemicals has attracted much attention as integrated triglyceride biorefinery technology [43–51]. Transesterification of triglycerides with methanol produces a mixture of glycerol and fatty acid methyl esters that can be commercially exploited as biodiesel, biolubricants, and biosurfactants. Glycerol can be converted to platform chemicals such as glycerol carbonate, propylene glycol, acrylic acid, and trioses (glyceraldehyde (GLA) and 1,3-dihydroxyacetone (DHA)). Trioses are also obtained by the retro-aldol fragmentation of fructose. In the presence of Lewis acids or bases, trioses are converted into lactic acid (LA) which is a bio-based commodity chemical with many applications, including the rapidly growing use of polylactate as a bioplastic.

**Figure 4.**
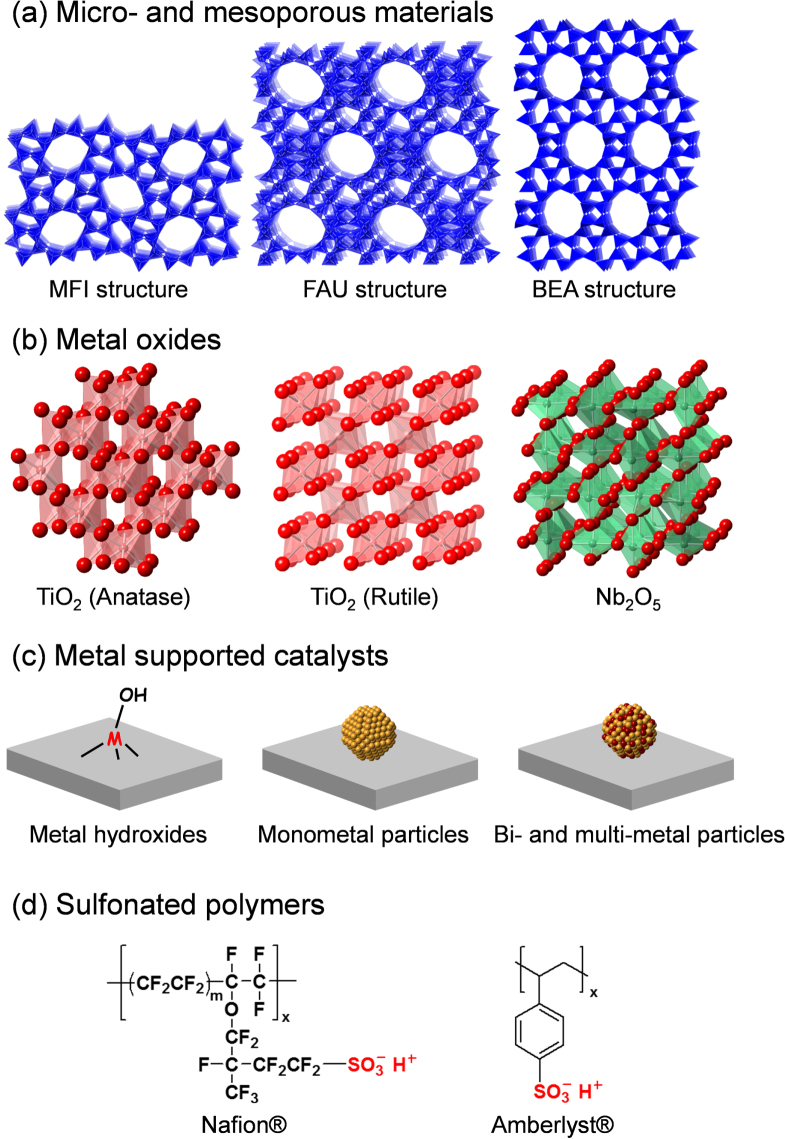
Examples of relevance to materials used in biomass conversion.

Various types of chemicals can be obtained from renewable biomass as a sustainable feedstock. Recently, numerous solid catalyst systems for the effective conversion of biomass feedstocks into high value-added chemicals and fuels have been developed. Solid catalysts are classified into four main groups with respect to their structures and substrate activation properties: (a) micro- and mesoporous materials, (b) metal oxides, (c) supported metal catalysts, and (d) sulfonated polymers (figure 4). This review article focuses on the activation of substrates and/or reagents on the basis of groups (a)–(d), including reaction mechanisms. In addition, recent progress in chemocatalytic processes for the production of five industrially important products (HMF, LA, GLA, DHA, and FDCA) as bio-based plastic monomers and their intermediates is comprehensively summarized. Details on the valorization of waste biomass by conversion to platform chemicals and fuels have been summarized in many excellent books and review articles [14–53]. Abbreviations of chemical compounds are summarized in table 1.

**Table 1.** Abbreviations of chemical compounds.

Abbreviation	Chemical compound
DFF	2,5-diformylfurane
DHA	1,3-dihydroxyacetone
FDCA	Furan-2,5-dicarboxylic acid
FFCA	5-formyl-2-furan-carboxylic acid
GLA	Glyceraldehyde
GLCEA	Glyceric acid
GLCOA	Glycolic acid
HA	Hydroxypyruvic acid
HFCA	5-hydroxymethyl-2-furan-carboxylic acid
HMF	5-hydroxymethylfurfural
LA	Lactic acid
PAL	Pyruvaldehyde
TA	Tartronic acid
THF	Tetrahydrofuran

## 2. Solid catalysts for biomass conversion into value-added chemicals

In this section, we focus on the structures and chemical properties of the solid catalysts (a)–(d), and present catalytic processes for biomass conversion. Some reaction mechanisms, including the interaction between substrates and active sites, are also described. There are two main advantages of solid catalysts for liquid-phase reactions over homogeneous catalysts: (i) facile recovery from the reaction solution and (ii) reusability without significant loss of activity. The definitive test for heterogeneity is to filter the catalyst during the course of the reaction at the reaction temperature and allow the filtrate to react further. If the reaction mixture is first allowed to cool to ambient temperature before filtration, leached metal ion(s) can re-adsorb onto the catalyst [54, 55]. Thus, the results for reuse experiments and leaching tests are also briefly summarized in tables 2–5 to discuss the heterogeneity and stability of each solid catalyst.

**Table 2.** Representative examples of the heterogeneously catalyzed formation of HMF from hexoses.

Entry	Catalyst	Substrate	Solvent	Temp. (K)	Time	Conv. (%)	HMF select. (%)	Comments	Reference
1^a^	Sulfonated resin (Dowex)	Fructose	Water/acetone	423	10 min	95	77	Reuse (5 times), requires microwave irradiation	[210]
2^b^	*γ*-Titanium phosphate	Fructose	Water	373	1 h	47	89	Reuse (2 times)	[148]
3^c^	c-Zirconium phosphate	Fructose	Water	373	1 h	52	86	Reuse (2 times)	[148]
4	TiO_2_ nanoparticle	Fructose	DMA	413	2 min	—	74	Reuse (5 times), requires microwave irradiation, addition of 10 wt% LiCl	[153]
5^d^	Amberlyst-15	Fructose	DMSO	393	2 h	100	100	Reuse (3 times)	[211]
6	Nafion NR50	Fructose	DMSO	393	2 h	100	94	Reuse (no)	[211]
7^e^	H-*β* zeolite	Fructose	DMSO	393	2 h	100	97	Reuse (no)	[211]
8^f^	WO_3_/ZrO_2_	Fructose	DMSO	393	2 h	100	94	Reuse (no)	[211]
9	H-*β* zeolite	Glucose	DMSO/THF/water	453	3 h	78	55	Reuse (4 times, regeneration by calcination at 823 K)	[127]
10	Sn-*β* zeolite/HCl	Glucose	Water/THF	453	70 min	79	72	Reuse (no), addition of excess NaCl	[126]
11	Ti-*β* zeolite/HCl	Glucose	Water/THF	453	105 min	76	70	Reuse (no), addition of excess NaCl	[126]
12	Sn-*β* zeolite/HCl	Glucose	Water/BuOH	453	90 min	75	55	Reuse (no), addition of excess NaCl	[126]
14	Phosphate/Nb_2_O_5_	Glucose	Water	393	2 h	92	52	Reuse (4 times)	[149]
15	Phosphate/TiO_2_	Glucose	Water/THF	393	2 h	98	83	Reuse (4 times)	[152]

a Dowex 50wx8-100 (Sigma-Aldrich, 50–100 mesh beads, gel, water content: 40–70%) consisting of a sulfonated copolymer of styrene and divinyl benzene in the hydrogen form (total exchange capacity of 1.7 meq mL^−1^).

b *γ*-phase titanium phosphate (Ti(PO_4_)(H_2_PO_4_)·2H_2_O) with interlayer distance of 1.16 nm.

c Cubic-phase zirconium pyrophosphate (c-ZrP_2_O_7_).

d Amberlyst-15 and Nafion resin were purchased from Sigma-Aldrich.

e H-BEA zeolite (JRC-Z-HB25, SiO_2_/Al_2_O_3_ = 25 ± 5) provided by the Catalysis Society of Japan.

f WO_3_-loaded ZrO_2_ prepared by impregnation of hydrated zirconia with an aqueous solution of ammonium paratungstate at pH 10, followed by calcination at 873 K.

**Table 3.** Representative examples of the heterogeneously catalyzed formation of LA/methyl lactate from trioses.

						Yield (%)			
Entry	Reactant	Catalyst	Solvent	Temp. (K)	Time (h)	Alkyl lactate	LA	Comments	Reference
1^a^	DHA	H-USY	H_2_O	398	24	—	71	Reuse (continuous flow reactor, steep decrease in activity with time)	[116]
	GLA				48		63		
2^b^	DHA	H-*β*	H_2_O	398	24	—	63	—	[116]
	GLA				48		60		
3^c^	DHA	H-ZSM-5	H_2_O	398	24	—	32	—	[116]
	GLA				48		30		
4^d^	DHA	H-MOR	H_2_O	398	24	—	39	—	[116]
	GLA				48		32		
5^e^	DHA	H-Mont.	H_2_O	398	24	—	46	—	[116]
	GLA				48		44		
6^f^	DHA	SO_4_^2−^/ZrO_2_	H_2_O	398	24	—	39	—	[116]
	GLA				48		41		
7	DHA	Sn-*β*	H_2_O	398	24	—	90	Reuse (decrease in activity, 21% yield for the first reuse)	[115]
8^g^	DHA	Sn-Si-CSM	H_2_O	383	6	—	76	Reuse (3 times, continuous decrease in activity), Sn leaching (yes)	[118]
9	DHA	Sn-*β*	MeOH	363	24	>99	—	Reuse (3 times, no decrease in activity)	[115]
10	DHA	Sn-*β*	EtOH	363	—	>99	—	Reuse and Sn leaching (not evaluated)	[117]
11^a^	DHA	H-USY	MeOH	398	24	96	—	Reuse (experiment in continuous flow reactor, gradual decrease in	[116]
	GLA				48	98			
12^b^	DHA	H-*β*	MeOH	398	24	42	—	—	[116]
	GLA				48	63			
13^c^	DHA	H-ZSM-5	MeOH	398	24	17	—	—	[116]
	GLA				48	19			
14^d^	DHA	H-MOR	MeOH	398	24	8	—	—	[116]
	GLA				48	10			
15^e^	DHA	H-Mont.	MeOH	398	24	29	—	—	[116]
	GLA				48	30			
16^f^	DHA	SO_4_^2−^/ZrO_2_	MeOH	398	24	17	—	—	[116]
	GLA				48	37			
17^g^	DHA	Sn-Si-CSM	EtOH	363	6	100	—	Reuse (3 times), Sn leaching (yes)	[118]
			dodecanol			83			
			tetradecanol			54			

a Si/Al = 6.

b Si/Al = 12.5.

c Si/Al = 11.5.

d Si/Al = 10.

e H-Mont. = montmorillonite in proton form.

f SO_4_^2−^/ZrO_2_ = sulfated zirconia.

g Sn-containing mesoporous silica (MCM-41) after carbon deposition within mesopores.

**Table 4.** Representative examples of the heterogeneously catalyzed oxidation of HMF to FDCA with O_2_^a^.

							Yield (%)					
Entry	Catalyst	Additive (equiv.)	Solvent	Temp.(K)	O_2_ (MPa)	Time (h)	FDCA	DFF	HFCA	FFCA	Comments	Reference
1	Ru(OH)_*x*_/CeO_2_	—	Water	413	0.25	6	38	12	50	—	Reuse (3 times)	[173]
2^b^	Ru(OH)_*x*_/MgAl_2_O_4_	—	Water	413	0.5	18	56	4	30	—	Reuse (4 times), leaching (Mg: 0.9%, Ru: 0.02%)	[174]
3^c^	Ru(OH)_*x*_/La_2_O_3_	—	[EMIm] [OAc]	373	3	5	48	—	12	—	Leaching (9.9%)	[175]
4	Au/CeO_2_	NaOH (4)	Water	338	Flow (1)	8	>99	—	—	—	Au/CeO_2_: reuse (3 times), leaching (no)	[176]
	Au/TiO_2_	NaOH (4)	Water	338	Flow (1)	8	>99	—	—	—		[176]
5	Pt/C	NaOH (2)	Water	295	0.68	6	79	—	21	—	Pt/C: reuse (3 times),	[177]
	Pd/C	NaOH (2)	Water	295	0.68	6	71	—	29	—	Au/C: leaching (no)	[177]
	Au/C	NaOH (2)	Water	295	0.68	6	8	—	92	—		[177]
6	Au/hydrotalcite	—	Water	368	Flow (0.1)	7	>99	—	—	—	Reuse (3 times), leaching (no)	[178]
7^d^	Au/TiO_2_	HTFA (18–21)	AcOH	403	1	3	—	—	—	78	—	[179]
8	Au/HY zeolite	NaOH (4)	Water	333	0.3	6	>99	—	—	—	Reuse (4 times)	[180]
9	Ru/C	—	Water	383	2	—	—	29	—	<1	Reuse (5 times, decrease in activity), leaching (no)	[181]
10^e^	Ag/OMS-2	—	IPA	438	1.5 (air)	6	—	99	—	—	Reuse (6 times)	[182]
11	Au–Cu/TiO_2_	NaOH (4)	Water	368	1	4.5	95	—	5	—	Reuse (5 times), leaching (≤2% after 5th reuse)	[183]
12	Pt–Bi/C	NaHCO_3_ (4)	Water	373	4 (air)	6	98	—	1	—	Reuse (5 times), leaching (<0.5%)	[184]
13^f^	Au_8_Pd_2_/AC	NaOH (2)	Water	333	0.3	2	99	—	1	—	Reuse (5 times), leaching (no)	[185]
14	Au–Pd/CNT	—	Water	373	0.5	12	94	—	—	2	Reuse (6 times)	[186]

a Additive (equivalent with respect to HMF).

b Formic acid was formed in 10% yield.

c [EMIm][OAc] = 1-ethyl-3-methylimidazolium acetate. Formic acid was formed in 30% yield.

d HTFA = trifluoroacetic acid.

e IPA = isopropyl alcohol.

f AC = activated carbon.

**Table 5.** Representative examples of the heterogeneously catalyzed oxidation of glycerol to trioses with O_2_^a^.

						Yield (%)						
Entry	Catalyst	Additive (equiv.)	Temp. (K)	O_2_ (MPa)	Time (h)	DHA	GLA	GLCEA	TA	GLCOA	Comments	Reference
1	Pd/C	NaOH (1)	333	0.3	3	—	4	45	8	—	Pd/C: leaching (no)	[187]
	Pt/C	NaOH (1)	333	0.3	3	—	13	47	3	—		[187]
	Au/C	NaOH (2)	333	0.5	3	—	—	84	5	—		[187]
2	Au/CeO_2_	NaOH (pH 12)	333	Flow	5	10	—	16	3	5	Reuse (no), leaching (yes)	[188]
3^b^	Au/C	NaOH (2)	333	0.6	2	19	—	60	5	10	Reuse (4 times, decrease in activity)	[189]
4^c^	Pt/C	—	333	Flow	6	10	3	47	—	7	Reuse (6 times)	[190]
5^d^	Pt/MWCNTs	—	333	Flow	8	2	—	56	—	3	—	[191]
6^e^	Pt/S-MWCNTs	—	333	Flow	6	12	1	62	—	5	Reuse (5 times, decrease in activity)	[192]
7^f^	Pt/SPDVB-dep	—	333	0.5	24	—	—	60	—	—	Reuse (4 times), leaching (yes)	[193]
8	Au–Pt/C	NaOH (pH 12)	333	0.1	—	17	—	17	—	—	—	[194]
9	Pt–Bi/C	—	343	Flow	2	42	2	10	—	—	—	[195]
10	Pt–Cu/C	—	333	Flow	6	9	1	61	—	8	—	[196]
11^g^	PtSb/MWCNTs	—	333	Flow	—	46	2	26	—	3	Reuse (5 times)	[197]
12^h^	Pd–Ag/C	—	353	0.3	24	44	1	3	—	1	Reuse (decrease in activity), leaching (Pd: 0.1%, Ag: 0.5%)	[198]
13^i^	Au–Pd–Pt/TiO_2_	—	373	0.3	4	13	—	20	<1	3	Reuse (2 times, decrease in activity), leaching <1%)	[199]

a Additive (equivalent with respect to glycerol). Solvent (water).

b C1 by-products such as CO_*x*_ and HCHO were formed in 7% yield.

c C1 by-products such as CO_*x*_ and HCHO were formed in 4% yield.

d MWCNTs = multi-wall carbon nanotubes. C1 by-products such as CO_2_, HCHO, and HCOOH were formed in 14% yield.

e S-MWCNTs = sulfur-treated multi-wall carbon nanotubes.

f Pt/SPDVB-dep = gas-phase sulfonated mesoporous polydivinylbenzene (SPDVB)-supported platinum catalyst.

g HA was formed in 5% yield.

h HA was formed in 1% yield.

i HA was formed in <1% yield.

### 2.1. Micro- and mesoporous materials

**Figure 5.**
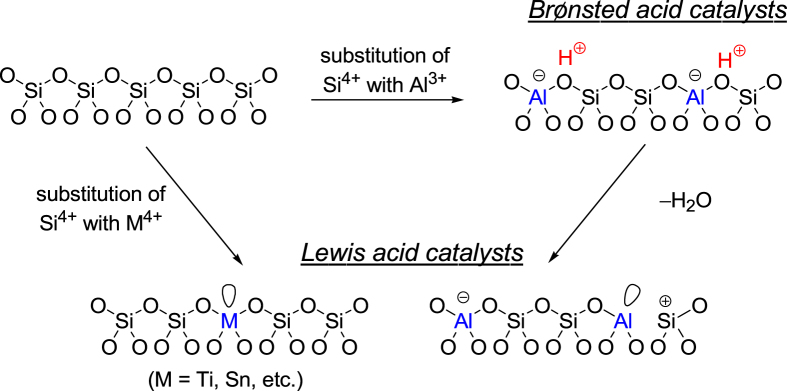
Mechanism for the formation of Br⊘nsted and Lewis acid sites within the zeolite framework.

Zeolites are an important class of inorganic crystalline materials that have been widely used in petroleum refining, and in the petrochemical and fine chemical industries as catalysts, adsorbents, and ion-exchangers [56–85]. The zeolite framework has an ordered distribution of micropores with diameters typically less than 2 nm. In comparison with other microporous materials, the zeolite framework is built exclusively from TO_4_ tetrahedra (T denotes tetrahedrally coordinated Si, Al, or P). Each TO_4_ tetrahedron is connected with four neighbors by sharing their vertex O atoms, which forms the three-dimensional four-connected zeolite framework. Although all zeolites are constructed from TO_4_ tetrahedra, they can be connected in different ways, which leads to the rich variety of zeolite structures. Figure 5 illustrates the mechanism for the formation of Br⊘nsted and Lewis acid sites within the zeolite framework. The substitution of Si atoms with trivalent Al atoms results in the formation of protons at the bridging O atoms (Si–OH–Al), and the evolved protons can act as Br⊘nsted acid sites. Acidic protons can be replaced by various metal cations (e.g., Na^+^, K^+^, Cs^+^, Mg^2+^, Ca^2+^, Ag^+^, Cu^2+^, Pd^2+^, Pt^2+^ etc) by ion-exchange. These metal ion-exchanged zeolites mainly work as base, oxidation, and hydrogenation catalysts depending on the nature of metal cations, and they are applied to a wide range of reactions including cracking of hydrocarbons, removal of nitrogen monoxide from emission of diesel engines, selective oxidation of methane and benzene, and synthesis of fine chemicals [56–85]. When Si atoms are replaced with high coordination number heteroatoms, the incorporated heteroatoms function as Lewis acid sites that can activate various nucleophiles. Some Lewis acid-doped zeolites exhibit remarkable activity, selectivity, and lifetime for multiple processes [86–88]. For instance, the development of TS-1 (a Ti^IV^-doped MFI-type zeolite, figure 4(a)) is viewed as one of the greatest breakthroughs in sustainable chemistry in the last few decades, having resulted in a greener processes that include the epoxidation of propylene with hydrogen peroxide (H_2_O_2_) [89]. In addition, Sn-*β* (Sn^IV^-doped BEA-type zeolite, figure 4(a)) has shown unparalleled activity and selectivity for the isomerization of glucose to fructose and the Baeyer–Villiger oxidation of ketones to lactones with H_2_O_2_ as the green oxidant [90–92]. In this section, the acid-catalyzed (i) formation of lactate from trioses and (ii) conversion of hexoses over micro- and mesoporous materials are described.

#### 2.1.1. Micro- and mesoporous materials for the formation of lactate from trioses

LA is currently emerging as a building block for a new generation of materials such as biodegradable plastics and solvents [93–95]. These new materials can be produced from biomass-derived precursors and have the potential to replace existing petroleum-based materials due to comparable or even superior properties [96]. LA also has the potential to become a central chemical feedstock in the chemical industry for the production of acrylic acid, propylene glycol, and various useful condensation products. Despite its high potential, the major obstacle to the wider implementation of LA-based materials and the use of an LA platform in the chemical industry is the high cost associated with the cumbersome manufacturing route of LA; large-scale production of LA relies on batch-wise fermentation of aqueous glucose under anaerobic conditions [97–102]. The fermentation reaction typically takes 2–4 days and requires continuous addition of calcium hydroxide to maintain a neutral pH level for optimal bacterial function, which results in the formation of calcium lactate. Crystallization of calcium lactate followed by acidification with sulfuric acid releases crude LA and gypsum. Typically, one ton of gypsum is formed for every ton of LA produced [93]. Further purification of LA is performed by esterification to methyl lactate followed by distillation and hydrolysis to release pure LA. Therefore, alternative chemocatalytic approaches toward the production of LA and its derivatives from abundant carbohydrates are of significant interest.

**Figure 6.**
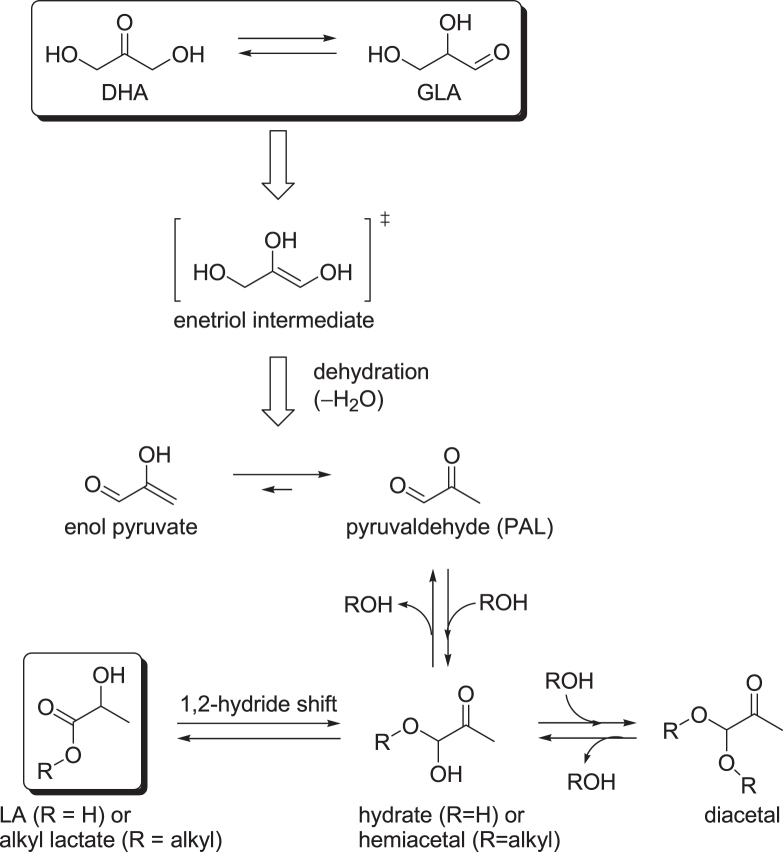
Reaction pathways for the conversion of trioses into lactate (alkyl lactate or LA) catalyzed by a Lewis acid catalyst [113].

The catalytic production of lactate from trioses (DHA and GLA) has been studied because the trioses can be obtained by aerobic oxidation of glycerol [103–106] or by fermentation using the Gluconobactor suboxydans strain [107–110]. Figure 6 represents the generally accepted mechanism for the conversion of trioses into alkyl lactates or LA. The two triose isomers of DHA and GLA easily interconvert according to a ketose–aldose isomerization, which shifts the carbonyl between C1 and C2. This reaction is, in principle, performed via an acid-catalyzed hydride shift, via a base-catalyzed mechanism with a proton shift (and intermediate enol), or via a concerted proton-coupled hydride shift in neutral media. The latter isomerization was recently studied in the presence of heterogeneous Lewis acid (Sn) by Roman-Leshkov and co-workers for hexose monosaccharides [90, 111, 112] and by Assary and Curtiss for trioses [113]. While LA formation from trioses starts with a dehydration step, it is still controversial as to whether DHA or GLA is the ultimate dehydrating substrate. Although the reaction rates are different, depending on the use of DHA or ALD, the conversion generally works well from both substrates. This is probably due to the rapid equilibrium between the two triose isomers. The dehydration reaction forms pyruvaldehyde (PAL) via an enetriol intermediate and enol pyruvate. PAL is prone to nucleophilic attack by water or an alcohol at the carbonyl carbon atom of the aldehyde to form a hydrate or hemiacetal, respectively. In alcoholic media, the hemiacetal can further react in two ways; the formation of a dialkyl acetal (in equilibrium with the hemiacetal) by reaction with another alcohol molecule or isomerization into an alkyl lactate. In water, only the latter isomerization to LA is possible. The isomerization and nucleophilic attack may also be concerted without the formation of intermediates. The isomerization step assumes a formal 1,2-hydride shift, which has been explained as an internal Cannizzaro reaction from isotope labeling experiments [114] or as a combination of Meerwein–Ponndorf–Verley (MPV) reduction and Oppenauer oxidation [115]. Both are formally indistinguishable, but either way, it was confirmed that the H atom of the aldehyde in PAL shifts to the adjacent carbon atom.

Two research groups independently investigated the use of H-USY zeolites (FAU-type structure, figure 4(a)) for the production of LA from trioses [114, 116]. They performed a screening with H-USY, H-ZSM-5, H-BEA and H-MOR along with a sulfated zirconia and H-montmorillonite, both in water and methanol. The H-USY zeolite with a bulk Si/Al ratio of 6 (H-USY(6)) outperformed all the other catalysts and also the other H-USY with a Si/Al ratio of 30 (H-USY(30)). Methyl lactate was obtained in 96% yield. Fourier transform-infrared spectroscopy (FTIR), using pyridine as a probe for acidity, combined with NH_3_-temperature programmed desorption (TPD) measurements showed that the ratio of Br⊘nsted over Lewis acid sites were 1.8 and 5.6 for H-USY(6) and H-USY(30), respectively. Working in water generally entailed lower rates, lower yields and also lower lactate selectivity. In addition, the zeolite catalyst in a promising continuous flow reaction setup was studied to evaluate the stability of the zeolite and its deactivation process. It was determined that LA destroys the catalyst structure, even at a concentration as low as 0.3 M. As expected, this is not observed for methyl lactate in methanol. Another cause for the deactivation of zeolite in water is catalyst coking, which is mainly attributed to PAL decomposition. An additional kinetic study with H-USY(6) in water revealed activation energy barriers of 53 and 61 kJ mol^−1^ for DHA dehydration and PAL to LA reaction, respectively. These values are clearly lower than those obtained with soluble Al^3+^ salts [115], while the rate-determining step is reversed, the hydride shift being the slowest step with the H-USY(6) zeolite.

Taarning and co-workers proved that particular Lewis acidic *β*-zeolites (BEA structure, figure 4(a)) were very active for DHA conversion [115]. A comparison of a series of zeolites with Ti-, Zr-, and Sn incorporated within the framework revealed a correlation between the catalytic activity and Lewis acid strength, with Sn being the strongest among the series. From a comparison of Al-*β* with a Br⊘nsted acidic ion-exchange resin yielding only dialkyl acetals, the authors also postulated that strong Br⊘nsted acidic zeolites are selective towards acetals, whereas Lewis acidic zeolites are selective towards alkyl lactates. The initial turnover rate (per Sn site) of methyl lactate formation with the Sn-*β* zeolite (45 mol Sn mol^–1^ h^–1^) was higher than that of homogeneous SnCl_4_·5H_2_O (4.2 mol Sn mol^–1^ h^–1^). Interestingly, steaming a parent Al-*β* zeolite to produce extra-framework Al enhanced the lactate yield with respect to the pristine H-Al-*β* [115]. However, the elaborate and time consuming synthesis procedure for Sn-*β* zeolite could hamper its large scale application. Alternative Sn-based catalysts or alternative synthesis routes to Sn-*β*, as very recently reported by Hermans and co-workers, may offer advantages in this respect [117].

**Figure 7.**
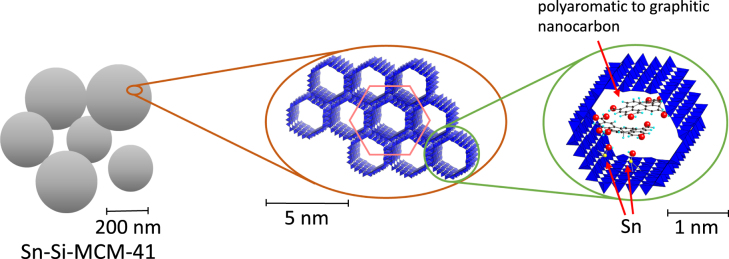
Schematic representation of the structure of carbon-deposited and Sn-incorporated mesoporous silica catalyst at various scale lengths [118].

Bifunctional catalysis for alkyl lactate synthesis from trioses has recently been reported by de Clippel *et al* [118]. Based on their previous work [119–121], they proposed a simple carbon–silica composite design to independently alter the number of Br⊘nsted acid sites from that of the Lewis acid sites (figure 7) [118]. Lewis acidity was imparted by grafting a mesoporous silica (MCM-41) with isolated Sn^IV^. Br⊘nsted acidity was introduced by polymerizing furfuryl alcohol in the mesopores followed by pyrolysis of the polymer to an active carbon phase. Oxygen-containing functional groups such as carboxylic acids and phenols acted as weak Br⊘nsted acid sites, as ascertained by the solid-state ^31^P magic angle spinning nuclear magnetic resonance (MAS-NMR) spectroscopy. The density of acid sites was controlled by the carbon loading, the degree of oxidation (dependent on the pyrolysis temperature) and an optional post-synthesis oxidation. Quantitative DHA conversion into ethyl lactate was achieved in ethanol with a composite containing 15% carbon that was pyrolyzed at 773 K and post-treated at 573 K under oxygen flow. This composite achieved a 7-fold increase of the initial turnover frequency (TOF) with respect to the parent Sn-MCM-41. The Sn atom is crucial for the selectivity by facilitating the 1, 2-hydride shift of PAL; however, it does not determine the reaction rate. The acid sites at the carbon surface accelerate the rate-determining dehydration step of DHA to PAL. Therefore, the catalytic potential of Sn in this reaction is evident when sufficient Br⊘nsted acidity is available in the closed environment. Catalyst regeneration for these composites was demonstrated by the observation of constant conversion and selectivity over three consecutive runs in ethanol. However, the importance of the spatial organization of the two catalytic sites was not examined in this study. The conversion of trioses into LA in water was also successful and a similar bifunctional catalysis explains the observed chemistry. However, the use of water entailed a decrease of activity in consecutive runs, due to deactivation (by carbonaceous deposits) of the catalyst, which is consistent with earlier reports for other catalyst types in water [116]. The composite was even capable of working in long-chain alcoholic media to afford long chain lactates in one step with high yields and interesting (e.g., solvent) properties [118].

#### 2.1.2. Micro- and mesoporous materials for conversion of hexoses

The isomerization of glucose into fructose is an important industrial reaction used mainly for the production of high-fructose corn syrup [122]. In recent years, glucose isomerization has played a crucial role in the synthesis of biomass-derived chemical platforms used for the production of fuels and chemicals [123]. The reaction is equilibrium-limited (*K*_eq_ ≈ 1 at 298 K), slightly endothermic (*ΔH*_r_ = 3 kJ mol^–1^), and typically catalyzed by an immobilized enzyme (xylose isomerase) [124]. Equilibrium mixtures often yield a product distribution of approximately 42% (w/w) fructose, 50% (w/w) glucose, and 8% (w/w) other saccharides [122]. Although fructose yields are high, the window of operation for this enzyme is very narrow and requires strict control over reactant purity, reaction temperature, and solvent pH. This significantly limits the cost-effective coupling of glucose isomerization with upstream and downstream biomass processing schemes such as cellulose hydrolysis and carbohydrate dehydration. In this respect, a robust heterogeneous inorganic catalyst would have clear advantages over the biological system.

Zeolites with the beta topology that contain tin (Sn-*β*) or titanium (Ti-*β*) metal centers in the framework are highly active catalysts for the glucose isomerization reaction in aqueous media. A 10 wt% glucose solution contacted with a catalytic amount of Sn-*β* at 383 K generated glucose, fructose, and mannose with respective yields of 46, 31, and 9% [90]. Similar product yields were achieved for glucose solutions with concentrations up to 45 wt%. Notably, the Sn-*β* catalyst exhibited high stability in that it did not show signs of deactivation after multiple cycles or after calcination, there was no leaching of Sn detected by elemental analysis; and a hot filtration test showed that the catalysis occurred heterogeneously. Most importantly, the Sn-*β* catalyst was able to promote the isomerization reaction in highly acidic aqueous environments with retention of activity and product distribution as in media without added acid. This feature enables Sn-*β* to couple isomerization with other acid-catalyzed reactions. The zeolite topology and the nature of the tin site were shown to significantly influence the catalytic activity. The isomerization reaction did not proceed with a medium pore zeolite (MFI structure, figure 4(a)), most likely because glucose molecules are not able to enter the smaller pores. Mesoporous stannosilicates (e.g., Sn-MCM-41) were active, while their activities were considerably lower than that of Sn-*β*. The reaction did not proceed when using SnO_2_, SnCl_4_, or SnO_2_-*β* (created by the incorporation of SnO_2_ nanoparticles into the pores of zeolite beta) as catalysts [90, 111].

**Figure 8.**
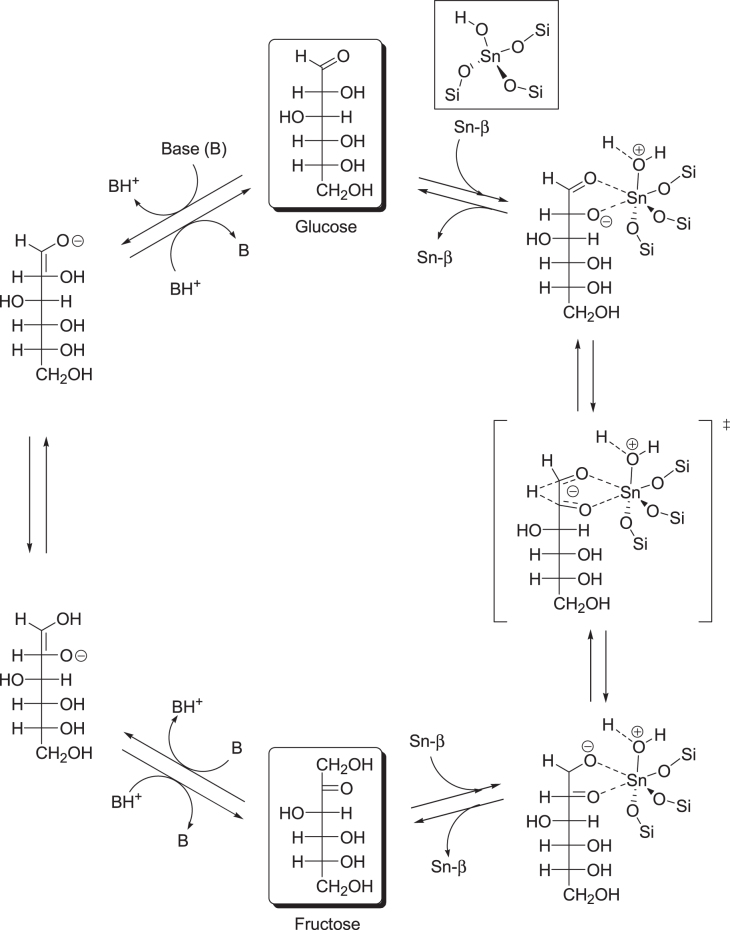
Possible reaction mechanism for Lewis acid (Sn-*β*) or Br⊘nsted base (NaOH) catalyzed transformation of glucose into fructose [111, 125].

These results indicate that isolated tin sites tetrahedrally coordinated to the crystalline zeolite framework are necessary for catalyzing the isomerization of glucose in aqueous media and that the degree of hydrophobicity surrounding the active sites is likely an important parameter to achieve the desired reactivity. A detailed NMR study revealed that Sn-*β* acts as a true Lewis acid during the isomerization of glucose in water [111]. Isotopically labeled glucose molecules were used to show that glucose isomerization with Sn-*β* proceeds via intramolecular hydride shift. This hydride shift pathway is similar to that observed in MPV reactions mediated by Lewis acids involving a six-membered transition state between the metal center, the carbonyl group, and the hydroxyl group in the sugar (figure 8) [111, 125]. In contrast, a similar spectroscopic study performed with labeled glucose and NaOH showed that the reaction proceeds via proton abstraction and an enolization pathway that is typically observed in base-mediated isomerization (figure 8) [111]. Replacing water with methanol as the solvent resulted in no isomerization activity for Sn-*β*, which is unexpected given that most Lewis acid catalysts perform better in the absence of water.

Nikolla *et al* published promising results for glucose dehydration with a bifunctional catalyst system of tin and titanium *β*-zeolites in combination with HCl [126]. An optimum HMF yield of 57% at 79% conversion was obtained from a 10 wt% glucose solution in 26 wt% aqueous NaCl solution at pH = 1 and in the presence of 0.5 wt% Sn-*β* and 3 equivalents (v/v) of tetrahydrofuran (THF) as an extraction solvent for 70 min at 453 K. When Ti-*β* was used, the yield was 53% at 76% conversion for 105 min. Sn-*β* maintained its activity, even in the presence of aqueous phases saturated with chloride salts, such as NaCl. In this case, the specific combination of Lewis and Br⊘nsted acid catalysts was shown to benefit glucose conversion toward HMF by efficiently performing a cascade reaction without excessive by-product formation. Dealuminated beta zeolite also could act as an effective bifunctional catalyst for direct transformation of glucose to HMF [127].

### 2.2. Metal oxides

Some metal oxides and phosphates have both Br⊘nsted and/or Lewis acid sites on the surface that can be catalytically active for various organic reactions [128, 129]. Representative examples are metal oxides and phosphates consisting of group IV and V elements. Niobic acid (Nb_2_O_5_·*n*H_2_O) is the most convenient precursor for the preparation of niobium pentoxide (Nb_2_O_5_, figure 4(b)), which is obtained upon complete dehydroxylation [130–133]. The presence of labile protons in Nb_2_O_5_·*n*H_2_O is responsible for its strong acidity, which corresponds to a 70% H_2_SO_4_ solution. Contrary to that observed with most acidic metal oxides, its acidity decreases with increasing pretreatment temperature [131, 132]. The dehydration of Nb_2_O_5_·*n*H_2_O occurs around 363–453 K, as revealed by thermogravimetric measurements in combination with differential thermal analysis [131]. Upon complete dehydroxylation (calcination above 773 K), niobium pentoxide exhibits very weak acidity, accompanied by a significant loss in surface area and a change from the amorphous to crystalline form. The Br⊘nsted acidity of Nb_2_O_5_·*n*H_2_O is very high, comparable to that of protonic zeolites according to ^1^H NMR results, and turns toward Lewis acidity following water elimination [131, 132]. Metal phosphates are well known for their acid properties, thus making them promising acid catalysts [128]. They are characterized by polymeric metal(IV) phosphates with layered structures, each layer consisting in a plane of tetravalent metal atoms sandwiched between planes of different hydrogen phosphate/phosphate species [134, 135], which contain both Br⊘nsted and Lewis acid sites [136, 137], the nature and concentration of which result is strictly related to the phase structures and thermal treatment history. In particular, zirconium and titanium phosphates belonging to this class of compounds have been used as acid catalysts for the dehydration of alcohols [138, 139], isomerization of olefins [140–143] and the rearrangement of terpenes [144].

Layered cubic-zirconium- and *γ*-titanium-phosphates [145–147] were used as acid catalysts in the dehydration of fructose to HMF in water [148]. When cubic zirconium pyrophosphate was employed in fructose dehydration, 86% HMF selectivity at 52% fructose conversion was obtained at 373 K for 1 h. Under analogous conditions, *γ*-titanium phosphate also exhibited promising performance (89% HMF selectivity at 89% fructose conversion). When the reaction products were extracted and the catalysts recycled, the performance was retained up to very high conversion.

**Figure 9.**
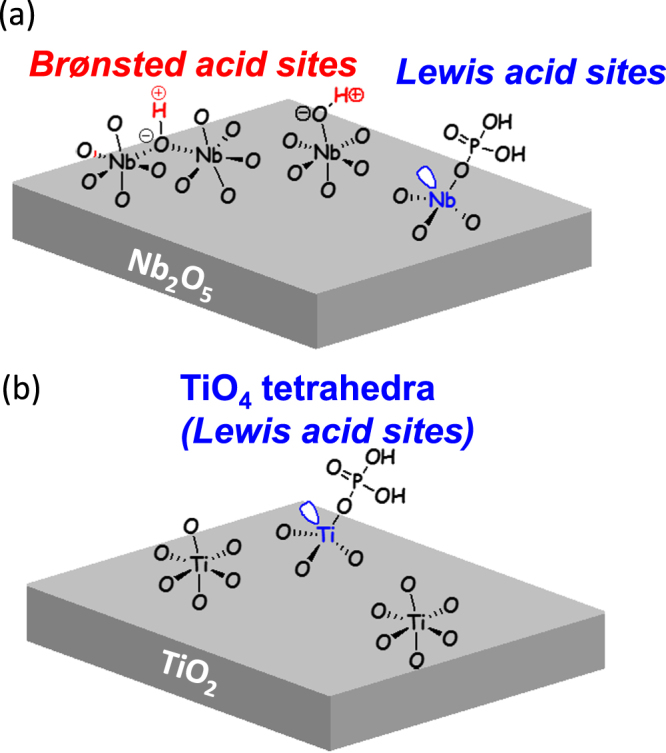
Representative structure of Lewis acid center on (a) phosphate/Nb_2_O_5_·*n*H_2_O and (b) phosphate/TiO_2_ [149, 152].

Phosphate-immobilized niobium oxide (phosphate/Nb_2_O_5_, figure 9(a)) has been reported to be an effective catalyst for direct HMF formation from glucose in water [149]. Phosphate/Nb_2_O_5_ produced HMF with 92% conversion and 52% selectivity, and no decrease in activity was observed even after several reuses of the catalyst. There is no difference in HMF formation between Nb_2_O_5_ and sodium-immobilized niobium oxide (Na^+^/Nb_2_O_5_) without Br⊘nsted acid sites, which indicates that HMF formation over Nb_2_O_5_ does not proceed on the Br⊘nsted acid sites. FTIR and Raman spectroscopies were used to investigate the Lewis acid sites on Nb_2_O_5_ in the presence of water, which indicated that NbO_4_ tetrahedra (Lewis acid sites) on Nb_2_O_5_ particle surfaces immediately form NbO_4_–H_2_O adducts in the presence of water. However, some adducts can still function as effective Lewis acid sites that catalyze the glucose to HMF transformation. Thus, some NbO_4_ tetrahedra present in insoluble Nb_2_O_5_ can act as Lewis acids, even in water (figure 9(a)).

Many metal oxides of the group 4 and 5 elements, including Nb_2_O_5_, are composed mainly of octahedral MO_6_ (M: metal) units with saturated coordination spheres (figure 9(a)), and polyhedral MO_*x*_ with unsaturated coordination spheres, such as tetrahedral MO_4_, also present on the surface. Unsaturated coordination MO_4_ tetrahedra act as Lewis acids, although MO_4_ species are considered to not function as effectively in water as other Lewis acids. NbO_4_ species in Nb_2_O_5_ acting as water-tolerant Lewis acid sites suggests that ubiquitous anatase TiO_2_ (figure 9(b)) with TiO_4_ species on the surface would also function as an insoluble, easily separable, and water-tolerant Lewis acid catalyst [149–152]. However, bare anatase TiO_2_ cannot act as an efficient heterogeneous catalyst for the selective transformation of glucose into HMF, which requires selective isomerization of glucose into fructose and intramolecular dehydration of fructose, because of intermolecular side reactions [152]. In contrast, TiO_2_ modified with H_3_PO_4_ (phosphate/TiO_2_, figure 9(b)), where OH groups on TiO_2_ are esterified into O-PO(OH)_2_ by phosphoric acid, exhibits high HMF yield (ca. 80%) in a THF–water mixture [152]. Such a high HMF yield can be achieved even with a diluted glucose solution (ca. 1 wt%) and high catalyst/glucose ratio (50/20 wt%). The original activity was maintained for subsequent reactions, which demonstrates that phosphate/TiO_2_ can function as a stable and reusable heterogeneous catalyst for HMF production. Self-assembled mesoporous TiO_2_ nanoparticulate material with well-defined nanospherical morphologies also efficiently catalyzed the dehydration of D-fructose and D-glucose into HMF in dimethylacetamide-LiCl solvent under microwave assisted heating [153].

### 2.3. Supported metal catalysts

Supported metal catalysts are one of the most important catalysts that have been utilized for many large-scale processes, including petroleum refining, vehicle exhaust gas scrubbing, and chemicals syntheses such as hydrogenation and oxidation [154–156]. Recently, catalysis by metal nanoparticles (especially Au) has attracted much global attention from researchers after reports by the Haruta group on the exceptional performance of Au nanoparticles supported on TiO_2_ for CO oxidation [157–163]. Control of the nanoparticle size, shape, and dispersity generally has a major effect on the reactivity and selectivity of these catalysts. In addition, the immobilization of metallic nanoparticles on a catalytically inert metal-oxide support often results in a dominant impact on the activity due to strong metal–support interactions that influence the electronic charge of the metallic catalysts. Not only monometallic nanoparticles, but also bi- and multi-metallic nanoparticles, have well investigated for many catalytic reactions, due to their beneficial catalytic properties that are typically different from those of the constituent metals, which result from electronic and/or geometric synergistic effects [164–167].

Much attention has been paid to the development of catalytic oxidation systems, and the choice of oxidant determines their practicability and efficiency. While many oxidants have been extensively investigated for catalytic liquid-phase oxidation processes, H_2_O_2_ and molecular oxygen (O_2_) are regarded as green oxidants due to their high content of active oxygen species, high atom efficiency, and production of only water as a by-product [89, 168–170]. The ideal system for greener and cleaner oxidation employs O_2_ together with a recyclable solid catalyst in a nontoxic solvent. In this section, liquid-phase oxidation (mainly in water) of (i) HMF to FDCA and (ii) glycerol to trioses, three-carbon acids, and two-carbon acids with O_2_ over supported metal catalysts are described.

**Figure 10.**
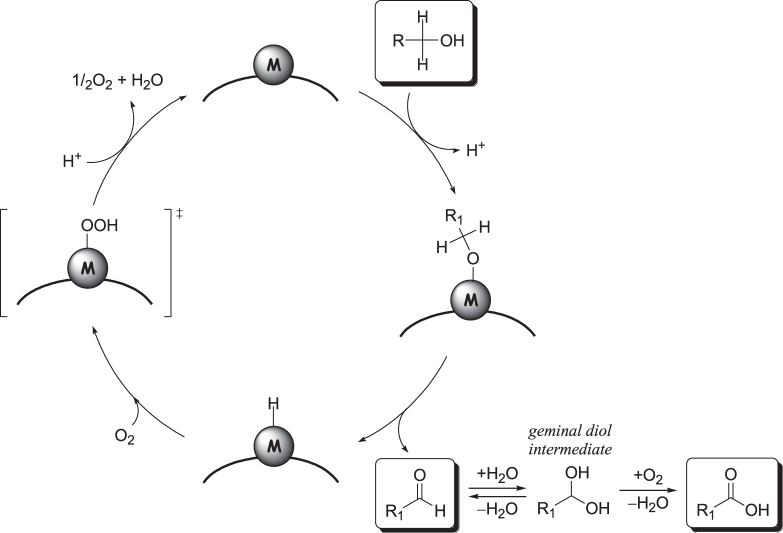
Proposed mechanism for the oxidation of a primary alcohol and successive oxidation of the corresponding aldehyde over a supported metal catalyst with O_2_ [171].

Both oxidation processes consist of two important reactions steps, (i) the oxidation of alcohols to carbonyl compounds such as aldehydes and/or ketones and (ii) subsequent oxidation of aldehydes to carboxylic acids. The proposed reaction mechanism for the oxidation of alcohols into carbonyl compounds over supported metal catalysts is shown in figure 10 [171]. The oxidation likely proceeds in three steps, the first of which involves adsorption of alcohol onto the metal surface to give an adsorbed metal alkoxide. Secondly, *β*-hydride elimination proceeds to produce a carbonyl species and a metal hydride. Lastly, the metal-hydride is oxidized by O_2_ to regenerate the metal surface. The oxidation of an aldehyde to carboxylic acid has been proposed to proceed through a geminal diol intermediate that is formed by the reaction of an aldehyde with water.

#### 2.3.1. Supported metal catalysts for oxidation of HMF to FDCA

**Figure 11.**
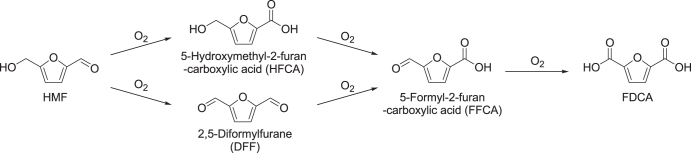
Reaction pathways for the catalytic oxidation of HMF to FDCA with O_2_.

To achieve highly selective oxidation of HMF into FDCA, the oxidation of two functional groups (aldehyde and alcohol) is required, the reaction pathways of which are shown in figure 11. The conversion of HMF into 5-formyl-2-furan-carboxylic acid (FFCA) proceeds via two possible intermediates of 2,5-diformylfurane (DFF; alcohol oxidation product) and 5-hydroxymethyl-2-furan-carboxylic acid (HFCA; aldehyde oxidation product), and the pathways are dependent on reaction conditions such as the type of metal and additives and the additive concentration (table 4 [172–184]). Supported Ru(OH)_*x*_ species are active for the aerobic oxidation of alcohols to carbonyl compounds [172]; thus, Riisager and co-workers reported HMF oxidation in water over Ru(OH)_*x*_ on various supports and under base-free conditions [173–175]. Among these catalysts, Ru(OH)_*x*_/CeO_2_ and Ru(OH)_*x*_/MgAl_2_O_4_ gave moderate yields of FDCA (38–56%) and could be reused 3–4 times without a significant loss of activity. The competitive formation of two intermediates (DFF and HFCA) was proposed from the reaction profiles. In contrast with HMF oxidation in water, the reaction in ionic liquids over Ru(OH)_*x*_/La_2_O_3_ showed significant leaching of Ru species out of the support and into the reaction solution [175].

The oxidation of HMF in water over supported monometallic nanoparticles such as Au, Pt, and Pd in the presence of 2–4 equivalents of NaOH with respect to HMF has been investigated [176, 177]. The base additive is required because (i) the base can facilitate dehydrogenation of hydroxyl groups and (ii) the amount of carboxylic acid adsorbed on the metal (especially Au) surface can be reduced. The reaction pathway under basic conditions has been proposed via HFCA and it is generally accepted that the alcohol oxidation step is slow in this reaction [171, 176]. Davis *et al* reported that the catalytic activity of Au/C was much higher than that for Pd/C and Pt/C, while the selectivity toward FDCA with Au/C (8%) was much lower than that with Pd/C and Pt/C (71–79%) [177]. The selectivity of supported Au catalysts toward FDCA can be increased to >99% by changing the carbon support to CeO_2_, TiO_2_, or HY zeolite [176, 180]. Corma and co-workers reported the efficient oxidation of HMF to FDCA over Au nanoparticles (3.5 nm) supported on CeO_2_; however, the catalytic activity of the recovered catalysts decreased under identical conditions [176]. Xu and co-workers reported that Au nanoclusters (1 nm) encapsulated within a HY supercage had higher catalytic activity than Au on other supports (TiO_2_, CeO_2_, Mg(OH)_2_, ZSM-5, and H-MOR) for the selective oxidation of HMF into FDCA [180]. The acidic OH groups inside the supercage can stabilize the Au nanoclusters and promote reaction activity by strong interaction between gold and the hydroxyl groups.

The types of additives, metal species, and supports have a strong influence on the selectivity for HMF oxidation. In the case of Au/TiO_2_, FFCA could be selectively obtained with 78% yield using trifluoroacetic acid (HTFA) as an additive instead of NaOH [179]. In contrast with supported Au, Pt, and Pd catalysts, the Ag-OMS-2 catalyst selectively gave DFF in 99% yield [182]. Investigation on the effect of Ag substitution in K-OMS-2 by temperature-programmed reduction and NH_3_/CO_2_-TPD analyses showed that the addition of Ag decreased the reduction temperature of K-OMS-2 and acidic sites that cause side reactions of HMF to undesired products resulted in high catalytic activity and selectivity toward DFF.

Bimetallic catalysts such as Au–Cu, Au–Pd, and Pt–Bi exhibit superior activity than the corresponding monometallic counterparts [183–185]. Hutchings and co-workers reported that well defined Au–Cu alloy nanoparticles (4.4 nm) supported on anatase TiO_2_ exhibited much higher activity and stability for HMF oxidation than their monometallic counterparts (i.e., Au/TiO_2_ and Cu/TiO_2_), possibly due to Au site isolation effects caused by alloying [183]. In addition, the Au–Cu/TiO_2_ catalyst could be easily recovered and reused without significant leaching or agglomeration of the metal nanoparticles. Similar effects of alloying on the activities and stabilities of Au_8_Pd_2_/AC and Pt–Bi/C have been reported [184, 185].

The base-free oxidation of HMF to FDCA has attracted considerable attention because HMF is not stable under basic conditions. However, most catalytic systems showed very low FDCA yields in the absence of a base, and thus highly efficient oxidation of HMF to FDCA under base-free conditions is a challenging research subject. Ebitani and co-workers reported that Au nanoparticles (3.2 nm) supported on basic hydrotalcite catalysts gave an excellent yield of FDCA without the addition of a homogeneous base such as NaOH and NaHCO_3_ [178]. A subsequent study by Riisager and co-workers showed that Mg^2+^ ions leached out of hydrotalcite (i.e., hydrotalcite acted as a stoichiometric base) during the oxidation reaction [174]. Recently, Wang and co-workers reported the stability and efficiency of Au–Pd nanoparticles (2–3 nm) supported on carbon nanotubes (CNTs) for the aerobic oxidation of HMF to FDCA in water without the addition of a base [186]. The functionalization of CNT surfaces plays an important role in the formation of FDCA, and CNTs that contain more carbonyl/quinone and less carboxyl groups favor FDCA formation by enhancing the adsorption of reactants and reaction intermediates.

#### 2.3.2. Supported metal catalysts for formation of trioses from glycerol

**Figure 12.**
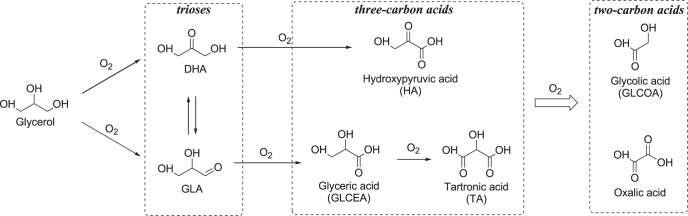
Reaction pathways for the catalytic oxidation of glycerol with O_2_.

Glycerol is a major by-product of biodiesel synthesis through the transesterification of triglycerides with methanol, as discussed in section 1.2 [43–51]. Glycerol oxidation to produce high value-added chemicals including trioses (DHA and GLA) and three-carbon acids (glyceric acid (GLCEA) and tartronic acid (TA)) could help biodiesel economics become more competitive with those of diesel produced from non-renewable resources. The possible reaction pathways for the oxidation of glycerol are shown in figure 12. Glycerol has two primary alcohols and one secondary alcohol; therefore, several oxygenated products are formed via multiple pathways. Oxidation of a primary or secondary alcohol in glycerol produces GLA or DHA, respectively, and these two products are in equilibrium when in aqueous solution, depending on the pH value. The sequential oxidation of GLA and DHA gives three-carbon acids such as hydroxypyruvic acid (HA), GLCEA, and TA, with successive oxidation of the three-carbon acids to C–C bond cleaved two-carbon acids such as glycolic acid (GLCOA) and oxalic acid. While many research groups have reported the oxidation of glycerol to carboxylic acids (e.g., HA, GLCEA, GLCOA) with O_2_ over heterogeneous catalysts, the selective oxidation of glycerol to carbonyl compounds (especially for GLA) is still limited (table 5 [187–199]). In this section, several examples of the selective oxidation of glycerol to trioses with O_2_ over supported metal catalysts are described.

In the presence of 1–2 equivalents of NaOH with respect to glycerol, selective oxidation of glycerol over supported Pd, Pt, and Au nanoparticles was investigated. Hutchings and co-workers investigated the oxidation of glycerol at 333 K with 0.3 MPa of O_2_ over Pd, Pt, and Au nanoparticles supported on graphite and carbon [187]. In all cases, GLCEA was the main product with very little GLA formed. The Pd/C and Pt/C catalysts gave respective GLA yields of 4 and 13%. Among the catalysts, Au/graphite or Au/activated carbon showed 100% selectivity to GLCEA with higher conversion than the Pt and Pd catalysts (70–97% selectivity to GLCEA). It has been proposed that NaOH aids initial dehydrogenation via H-abstraction of one of the primary OH groups of glycerol. An investigation on the effect of the support on glycerol oxidation over supported Au catalysts showed that a carbon support was the best [188] and that the oxygen-free carbon supports would influence electron mobility to and from the gold surface, which probably promotes both adsorption and the regeneration of hydroxide ions to result in high catalytic performance.

While the Au/C catalyst was inactive for glycerol oxidation in the absence of NaOH [187], the supported Pt catalysts were active under base-free conditions. Zhaoyin and co-workers reported that Pd nanoparticles (3.1 nm) supported on carbon achieved a GLCEA yield of 47% and that the catalyst was reused six times without significant loss of catalytic activity [190]. The GLCEA yield could be increased to 56–62% by changing the carbon support to multi-wall carbon nanotubes (MWCNTs) [191], sulfur-treated multi-wall carbon nanotubes (S-MWCNTs) [192], and gas-phase sulfonated mesoporous polydivinylbenzene (SPDVB) [193], while the catalytic activity or leaching of the Pt species out of the support could be decreased.

In a similar way to HMF oxidation to FDAC, bi- and multi-metallic catalysts such as Pt–Cu [196] and Au–Pd–Pt [199] exhibit superior activity than the corresponding monometallic counterparts. Hutchings and co-workers reported that the trimetallic Au–Pd–Pt/TiO_2_ was formed by the addition of Au to the best bimetallic catalyst, Pd–Pt/TiO_2_. [199] The TOF of the trimetallic Au–Pd–Pt/TiO_2_ was 378 h^–1^ and this value was 1.8 times larger than that for the bimetallic Pd–Pt/TiO_2_ catalyst (210 h^–1^) with retention of the selectivity towards C_3_ products. However, the Au–Pd–Pt/TiO_2_ catalyst was not reusable and small amounts of metal were leached, and significant particle agglomeration was observed.

Not only catalytic activity but also chemoselectivity could be controlled by alloying. Bimetallic catalysts of Pt–Bi/C [195], PtSb/MWCNTs [197], and Pd–Ag/C [198] showed much higher chemoselectivity toward DHA than Pt/C and Pd/C. Kimura *et al* reported that the Pt–Bi catalysts had high selectivity (ca. 50% at 20% glycerol conversion) toward DHA in a semibatch reactor [103], and the highest DHA yield of 48% at 80% conversion was reported by Varma and co-workers [195]. Hirasawa *et al* reported that Pd–Ag/C showed higher selectivity toward DHA and higher activity than Pd/C, where the DHA yield reached 44% at 52% glycerol conversion over Pd–Ag/C (Ag/Pd = 1) [198]. The Pd–Ag alloy phase is required to achieve high activity and selectivity toward DHA. The mechanistic investigation suggested that the terminal OH group of glycerol is adsorbed on the Ag site and the neighboring secondary OH group (CH–OH) is attacked by an oxygen species that is dissociatively adsorbed on the Pd site [200].

### 2.4. Sulfonated polymers

Commercially available polymeric ion-exchange resins have been used for a range of industrially important transformations [201]. The two main classes of ion-exchange resins are based on styrene-based sulfonic acids (Amberlyst® and Dow type resins, figure 4(d)), which exhibit very high activity for esterification and etherification, and the perfluorosulfonic acid-based catalysts, including the recently developed Nafion® resin/silica nanocomposites [202–206]. Nafion® resin is a perfluorinated resin-sulfonic acid and is a copolymer derived from tetrafluoroethylene and perfluoro-2-(fluorosulfonylethoxy)propyl vinyl ether. After hydrolysis of the sulfonyl fluoride, it yields the strongly acidic terminal -CF_2_CF_2_SO_3_H group (figure 4(d)) [201, 202]. These resins have very high activity for the formation of linear alkyl benzene, isomerization, and some select acylation type reactions [201–206]. Typical Br⊘nsted acids including these resin catalysts are not applicable to various sugar conversion reactions, because Lewis acid catalysts exhibit much higher catalytic performance for hydride transfer (glucose–fructose and PAL–LA isomerization step) in water than the Br⊘nsted acids [207–209]. In addition, Br⊘nsted acids can decompose HMF into two organic acids (levulinic acid and formic acid) in water, thus requiring continuous extraction of HMF from the reaction solution by an organic solvent or the stabilization of HMF by an appropriate solvent toward subsequent acid-catalyzed decomposition [207–209]. In this section, two representative examples of Br⊘nsted-acid catalyzed HMF formation from fructose are briefly described.

The catalytic dehydration of fructose into HMF by microwave heating was studied in acetone–water mixtures in the presence of a cation-exchange resin (Dowex 50wx8-100) catalyst [210]. The reaction in acetone–water reaction media at 423 K gave HMF in 73% yield at 94% conversion. No decrease of catalytic activity or selectivity was observed for five reuses of the resin, as determined by elemental analysis, which showed that sulfonic acid groups attached to the resin were stable under the experimental conditions. The comparison between conventional heating and microwave heating revealed that the latter has a significant acceleration effect, not only on fructose conversion, but also on the HMF yield. Fructose conversion and HMF yields (92 and 70%, respectively) by microwave heating were higher than those by sand bath heating (22 and 14% respectively). Shimizu *et al* developed a simple method for the effective production of HMF from fructose with various solid acid catalysts in dimethyl sulfoxide (DMSO), that was based on the continuous removal of water from the reaction mixture under reduced pressure at 0.97 × 10^5^ Pa [211]. The removal of water was effective for suppression of two undesired reactions; (i) the hydrolysis of HMF to levulinic acid and (ii) the reaction of partially dehydrated intermediates to condensation products. Nafion® resin and Amberlyst-15 gave high HMF selectivity with complete fructose conversion by the continuous removal of water from the reaction system.

## 3. Conclusions and future opportunities

Renewable biomass has attracted significant attention as a sustainable feedstock, and solid catalysts play an important role in the achievement of chemocatalytic processes for the manufacture of high value-added products such as biofuels, commodity chemicals, and new bio-based materials such as bioplastics. Various types of solid catalysts including micro- and mesoporous materials, metal oxides, supported metal catalysts, and sulfonated polymers can catalyze the conversion of biomass feedstocks into value-added chemicals and fuels. Heterogeneous Br⊘nsted and Lewis acid catalysts such as metal-substituted zeolites, surface-modified metal oxides, and cation-exchange resins are intrinsically effective for the hydrolytic conversion of cellulose and hemicellulose into C5 and C6 monosaccharides and their subsequent transformation into chemicals such as polyols, furans, and acids. Supported metal catalysts are also very useful and are most frequently studied for O_2_-based oxidation of HMF to FDCA and glycerol to trioses. In particular, bi- and multi-metallic catalysts exhibit superior activity and stability compared to the corresponding monometallic counterparts.

Although most solid catalysts can be easily recovered by simple filtration or centrifugation after the reaction and recycled without appreciable loss of their catalytic performance, successful examples of completely recovered and recycled catalysts are still limited. In addition, severe reaction conditions, such as hydrothermal conditions and high oxygen pressure, require a high ratio of additives, and microwave irradiation can sometimes cause instability and ion leaching of the solid catalysts. Therefore, future targets in this area will require novel catalyst design strategies to overcome these problems. Such concepts will open up a new avenue for the development of solid catalysts that are workable under mild reaction conditions for practical biomass conversion including challenging reactions such as high-yield direct syntheses of value-added chemicals from waste biomass as raw materials, and selective conversion of lignin into useful aromatic components. The density (i.e., quantity in a certain place) of lignocellulose is much lower than that of crude oil and natural gas, and the properties of biomass feedstocks are very much dependent on their sources; therefore, biorefinery processes cannot replace petroleum refinery processes at present. Our own impression is that other technological aspects, including collection and transport, should also be discussed to establish truly efficient biorefinery processes.
